# Consumer behaviour and experiences in a naturalistic online grocery store: implications for nutrition research

**DOI:** 10.1017/jns.2023.21

**Published:** 2023-03-14

**Authors:** Anna H. Grummon, Anna Claire Tucker, Violet Noe, Pasquale E. Rummo, Carmen E. Prestemon, Marissa G. Hall, Lindsay M. Jaacks, Veronica Lippuner, Lindsey Smith Taillie

**Affiliations:** 1 Department of Pediatrics, Stanford University School of Medicine, Palo Alto, CA, USA; 2 Department of International Health, Johns Hopkins Bloomberg School of Public Health, Baltimore, MD, USA; 3 Department of Nutrition, University of North Carolina Gillings School of Global Public Health, Chapel Hill, NC, USA; 4 Department of Population Health, New York University Grossman School of Medicine, New York, NY, USA; 5 Carolina Population Center, University of North Carolina Chapel Hill, Chapel Hill, NC, USA; 6 Department of Health Behavior, University of North Carolina Gillings School of Global Public Health, Chapel Hill, NC, USA; 7 Lineberger Comprehensive Cancer Center, University of North Carolina Chapel Hill, Chapel Hill, NC, USA; 8 Global Academy of Agriculture and Food Systems, University of Edinburgh, Midlothian, UK

**Keywords:** Acceptability, Feasibility, Grocery shopping, Nutrition intervention, Online grocery stores

## Abstract

Naturalistic online grocery stores could provide a novel setting for evaluating nutrition interventions. In 2021–2022, we recruited US adults (*n* 144, 59% low-income) to complete two weekly study visits: one in a naturalistic (‘mock’) online grocery store developed for research and one in a real online grocery store. Participants selected groceries and responded to survey questions. Analyses examined survey responses and expenditures on fifteen food categories (e.g., bread, sugar-sweetened beverages). Nearly all enrolled participants completed both visits (98% retention). Moreover, nearly participants all reported that their selections in the naturalistic store were similar to their usual purchases (95%) and that the naturalistic store felt like a real store (92%). Participants’ spending on food categories in the naturalistic store were moderately-to-strongly correlated with their spending in the real store (range of correlation coefficients: 0⋅36–0⋅67, all *P*-values < 0⋅001). Naturalistic online grocery stores may offer a promising platform for conducting nutrition research.

## Introduction

Online grocery shopping has increased dramatically in recent years. Online food and beverage sales in the US nearly tripled from 2017 to 2021, from $12⋅7 billion to $34⋅2 billion, and now comprise 10% of all US grocery sales^(1)^. Online grocery shopping is expected to continue to grow, with industry projections estimating that by 2024–2026, Americans will spend more than $47⋅6 billion on online groceries, or approximately 20% of all grocery sales^(1)^. Although online grocery shopping is more popular among higher-income households, online grocery shopping is growing rapidly among all demographic groups^(2)^, and policymakers and retailers are actively seeking to expand access to online groceries to new populations^(3–5)^.

As online grocery shopping becomes more popular, it will be important to understand how policies and interventions (from marketing practices^(6)^ and restrictions^(7)^ to warning labels^(8–11)^ to corporate initatives^(12,13)^) implemented in online stores affect consumer behaviour in this setting. Randomised trials are important tools for determining the causal impact of nutrition interventions on behaviour. However, conducting trials with real-world online grocers is not always feasible because retailers are often not willing or able to randomly assign consumers to different interventions. To address these challenges, we developed a naturalistic online grocery ordering platform designed to mimic a real-world online shopping experience while allowing researchers to implement nutrition interventions^(14)^. We have previously described the development of this store (see details below)^(14)^, but little is known about how consumers behave in and respond to naturalistic online grocery stores. Without information on consumer experiences in these settings, it is difficult to know whether naturalistic online grocery stores are a feasible, acceptable, and potentially ecologically valid venue for conducting nutrition research.

To address these gaps, the objectives of the present study were to (1) examine the extent to which participants found the naturalistic online store to be realistic and acceptable and (2) describe participants’ food and beverage selections in the naturalistic online grocery store and examine the extent to which these selections mirror their choices in a real online grocery store.

## Methods

### Participants

From November 2021 to March 2022, study staff recruited participants using Craigslist advertisements. To be eligible, participants needed to be aged ≥18 years, live in the US, be able to read and write in English, have shopped for groceries online during the past 12 months, and have access to the Internet (see Supplementary Table S1 for the eligibility screener). Supplementary Figure S1 shows the number of people screened, enrolled, and included in analyses.

### Setting

**Fig. 1.**
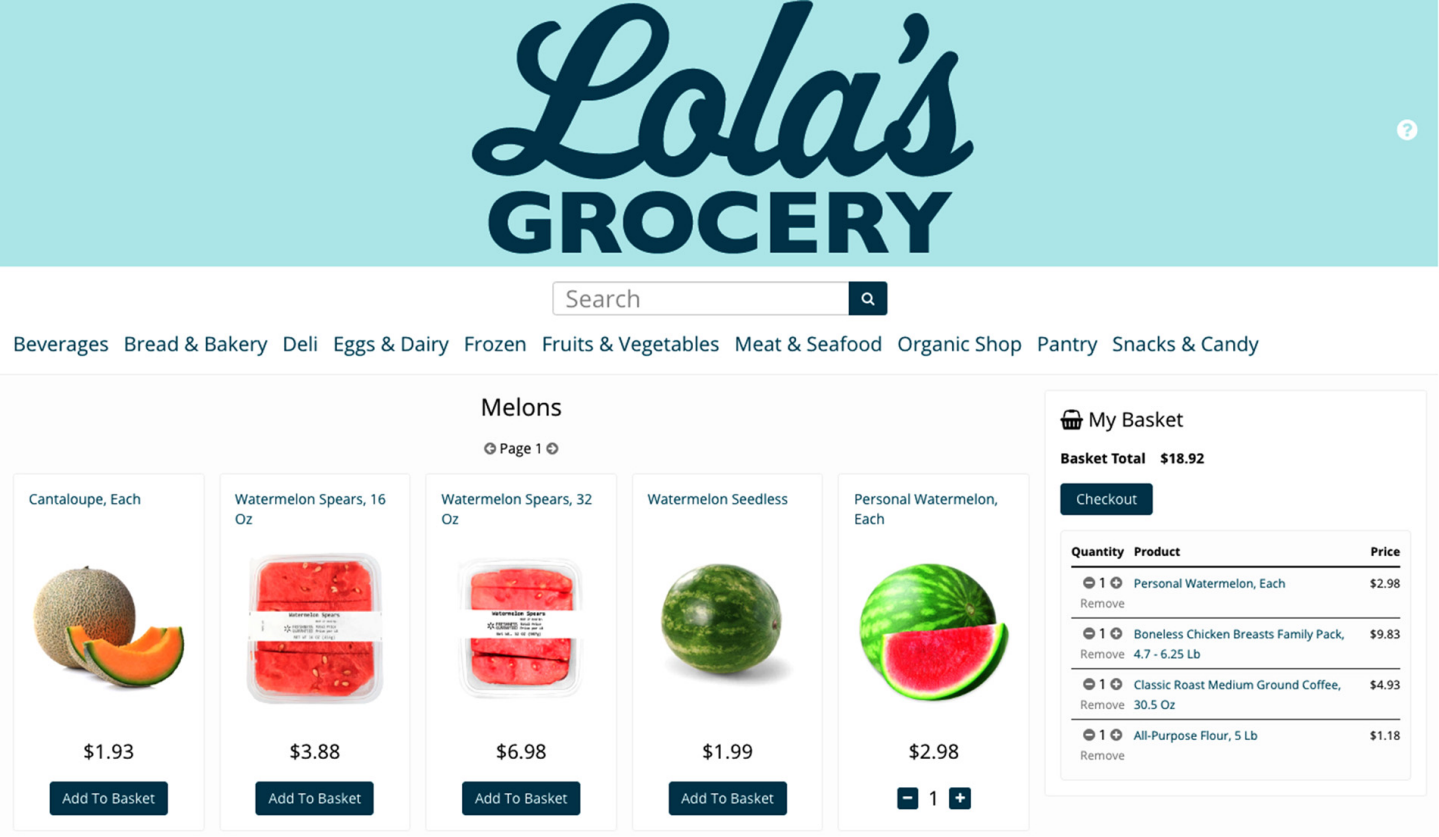
Sample page from the naturalistic online grocery store.

Participants completed online study visits with a member of the research team using the videoconferencing software Zoom. At each visit, participants completed a shopping trip in one of two stores: a previously developed naturalistic (‘mock’) online grocery store (Lola's Grocery) or a popular real online grocery store (Walmart.com). Details on the development of the naturalistic store have been published previously^(14)^. Briefly, the naturalistic store mimics the appearance and functionalities of real online grocers, including browsing, search, product pages, sorting and filtering, and checkout. It offers approximately 23 000 products sold at a top US online grocery retailer^(14)^, with products shown with images, price, and nutrition information. Researchers can manipulate aspects of the store (e.g., changing product prices, display order, and labels). Fig. 1 depicts an example page from the naturalistic online store.

### Procedures

Participants completed two online study visits (~30–60 min each) separated by ~1 week. During the first visit, research staff obtained informed consent from participants using an electronic survey. Additionally, during the first visit, research staff instructed participants to make a list of foods and beverages they typically buy each week when grocery shopping because most US households shop for groceries using a list^(15,16)^. Participants made the list using an online document (a Google Doc); study staff saved this list as a PDF and shared it with participants via Zoom chat immediately before both shopping tasks so participants could reference it while they shopped, if they chose.

During both study visits, participants selected foods and beverages in the naturalistic online store or the real online store and responded to survey questions about their experience shopping in that store. Store order (naturalistic *v*. real) was randomised within participants. Before the participants began shopping, research staff provided them with the following instructions: to select foods and beverages they typically buy each week using their list. The same instructions were used for both the naturalistic and the real online store. Participants then shopped on their own (i.e., without screensharing or involvement from the research team); one research staff member remained in the Zoom room with video and microphone off while the participant shopped to troubleshoot any technical difficulties in real time. For the shopping trip in the naturalistic online store, participants navigated to the naturalistic online store using a link provided by study staff. They placed items they wished to select in their cart. No payment was required. When participants were finished shopping, they were automatically re-directed to an online survey programmed in Qualtrics, which they completed on their own. The naturalistic online store automatically captured data on all products selected by participants, including information on product name, brand, quantity, and price. For the shopping trip in the real online store, participants navigated to the store's online grocery ordering platform using a link provided by study staff and logged into the study team's account. The account was pre-set to display items available at a St. Louis, MO store (selected because the grocery cost of living in St. Louis is similar to the US average^(17)^ and because prices in the naturalistic store were set using St. Louis grocery prices^(14)^). When they were finished shopping, participants responded to a survey programmed in Qualtrics, which they completed on their own. No payment was required. Research staff then logged into the study account and recorded the items in the participant's cart, including name, brand, quantity, and price. Because data collection coincided with frequent product shortages (e.g., stemming from the COVID-19 pandemic), participants notified staff of items they wished to purchase that were out of stock; staff recorded these in real time.

Participants received a $30 electronic gift card for completing the first visit and a $50 gift card for the second visit. The University of North Carolina and Harvard Longwood Campus Institutional Review Boards approved all study procedures.

### Measures

We assessed participants’ experiences in the store using survey measures. Survey measures included acceptability of the stores (e.g., satisfaction with number of options), acceptability of the study protocol (e.g., willingness to participate in another research study using the stores), and realism of the stores (e.g., extent to which the stores felt real) (Supplementary Table S2). Participants responded to these measures after both shopping trips (i.e., for both the naturalistic and real stores).

We also examined participants’ spending in both stores on fifteen food and beverage categories that are often targeted by food policies or are relevant for health (e.g., sugar-sweetened beverages, fruits and vegetables; Supplementary Table S3). Participants’ selections were categorised using product descriptions and nutrition information. Because the naturalistic online store did not offer alcohol, we excluded alcohol selections from the real online store from our analyses (five items selected by three participants).

### Analysis

To characterise acceptability, we estimated the proportion of participants who endorsed positive statements about their experience in the naturalistic online store. We also examined these outcomes for the real online store to provide a baseline against which the naturalistic store could be compared.

Next, we calculated means and SDs of expenditures in each food category for each store. To understand whether participants shopped similarly in the two stores, we calculated Pearson's correlation coefficients (*r*s), comparing expenditures in each category from the naturalistic online store to the real store. Correlations of 0⋅30–0⋅49 and ≥0⋅50 were considered moderate and strong, respectively^(18–20)^. We also examined whether expenditures differed between the naturalistic and real stores using paired *t* tests. We focused on proportional expenditures in each category to address potential inflation in the real online store that would not have occurred in the naturalistic store (which kept fixed prices throughout the study); we also report absolute expenditures (i.e., dollars) to provide context for estimates of proportional expenditures. All analyses were conducted in 2022–2023 using Stata MP version 17.1 (StataCorp LLC, College Station, TX, USA).

## Results

The average age in the sample was 39⋅5 years (sd = 11⋅0). The sample was diverse in terms of race/ethnicity, education, and income (Table 1). For example, 43% of participants identified as white, 38% as Black or African American, and 13% as Latino(a) or Hispanic. About one-third (30 %) had educational attainment of some college or less and more than half (59%) reported a household income of less than 200% of the Federal Poverty Level.

**Table 1.** Sample characteristics, *n* 144 US adults

Characteristic	%	*N*
Age		
18–29 years	19	27
30–39 years	38	55
40–49 years	25	36
50 years or older	18	26
Gender		
Woman	69	99
Man	29	41
Non-binary or another gender	2	3
Latino(a) or Hispanic	13	18
Race		
White	43	62
Black or African American	38	55
American Indian or Alaska Native	1	1
Asian or Pacific Islander	6	9
Other or Multiracial	11	16
Education		
High school diploma or less	9	13
Some college	21	30
College graduate or associates degree	54	77
Graduate degree	16	23
Household income, annual		
$0 to $24 999	10	14
$25 000 to $49 999	22	32
$50 000 to $99 999	43	62
$100 000 or more	25	36
Income less than 200% federal poverty level	59	85
Usual weekly grocery budget		
$100 or less	37	53
$101 to $150	26	38
$151 to $200	21	30
$201 or more	16	23
Household size		
1	20	28
2	24	34
3	26	37
4 or more	31	44
Number of children		
0	52	75
1	22	32
2	15	22
3 or more	10	14
Frequency of shopping for groceries online		
1 time per month or less	15	21
2–3 times per month	31	45
1 time per week	24	34
More than 1 time per week	31	44
Amount of household's shopping done by participant		
Half or less	6	9
More than half	13	19
All	81	116

*Note*. Missing data ranged from 0⋅0% to 0⋅7%.

Results suggested the study protocol was feasible. Study staff enrolled 157 participants in ~4 months (mid-November to early March, excluding winter holidays). Retention was high: of the 157 participants who were enrolled and completed their first study visit, 154 (98%) completed the second visit (Supplementary Figure S1).

**Fig. 2.**
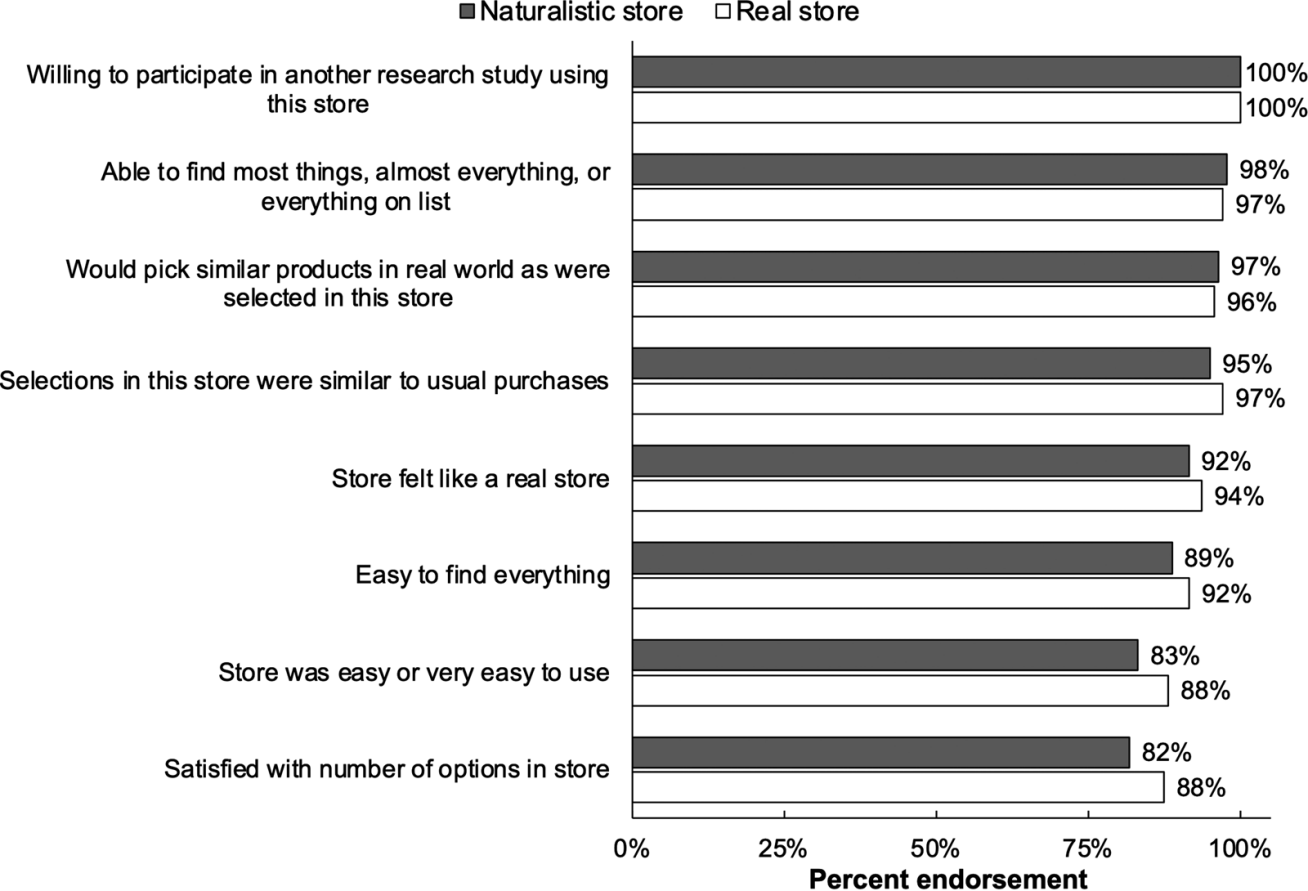
Acceptability of the naturalistic and real online grocery stores, *n* 144 US adults.

Participants’ survey responses indicated that the naturalistic online store offered a highly acceptable setting for nutrition research. For example, all participants (100%) indicated they would be willing to participate in another research study that required shopping in the naturalistic online store (Fig. 2). Likewise, nearly all (98%) reported they were able to find most things, almost everything, or everything on their shopping list in the naturalistic store. Most (83%) reported that the naturalistic online store was easy or very easy to use. Nearly all participants reported that they would pick similar products in the real world as they selected in the naturalistic online store (97%), that their selections in this store were similar to their usual purchases (95%), and that the naturalistic online store felt like a real online store (92%). On all process evaluation dimensions, the naturalistic online store performed similarly to the real store (Fig. 2).

Mean total spending in the online naturalistic store was $139⋅02 (sd = 83⋅96), similar to the real store (mean = $156⋅04, sd = 85⋅75) (Supplementary Table S4). Across food categories, meat and seafood had the highest absolute mean spending in both stores (approximately $30–32) while eggs had the lowest (approximately $2–3).

Analyses found moderate to strong correlations between spending on food categories in the naturalistic online store compared with spending in the real online store (range of correlation coefficients, *r* = 0⋅36–0⋅67, all *P-*values < 0⋅001, Table 2). The highest correlations were observed for meat and seafood (*r* = 0⋅67) and bread (*r* = 0⋅67), followed by dairy (*r =* 0⋅66) and fruits, vegetables, and legumes (*r* = 0⋅64) (all *P*-values < 0⋅001). There were few significant differences in mean expenditures on the fifteen food categories between the two stores; the only exceptions were that participants spent slightly less on eggs (1⋅6% *v*. 1⋅9%, *P* = 0⋅02) and salty snacks (4⋅6% *v*. 6⋅3%, *P* = 0⋅001) in the naturalistic store than the real store and slightly more on low-calorie drinks and juice (9⋅7% *v*. 7⋅4%, *P* = 0⋅002).

**Table 2.** Comparison of proportional expenditures on fifteen food and beverage categories in a naturalistic online store and a real online store, *n* 144 US adults

Category	Proportion of total expenditures					Correlation in spending between stores	
	Naturalistic online store mean (sd)		Real online store mean (sd)		*P*-value for difference in means	Pearson's *r*	*P*-value
Bread	3⋅7 %	(2⋅7 %)	3⋅8 %	(3⋅3 %)	0⋅62	0⋅67	<0⋅001
Cereal	2⋅2 %	(2⋅8 %)	2⋅5 %	(3⋅0 %)	0⋅24	0⋅54	<0⋅001
Dairy	7⋅0 %	(5⋅1 %)	6⋅4 %	(4⋅6 %)	0⋅12	0⋅66	<0⋅001
Eggs	1⋅6 %	(1⋅7 %)	1⋅9 %	(1⋅6 %)	0⋅02	0⋅49	<0⋅001
Frozen and packaged entrees	8⋅3 %	(8⋅0 %)	9⋅2 %	(10⋅7 %)	0⋅30	0⋅49	<0⋅001
Fruits, vegetables and legumes	20⋅0 %	(10⋅8 %)	18⋅7 %	(10⋅2 %)	0⋅08	0⋅64	<0⋅001
Low-calorie drinks and 100 % juice	9⋅7 %	(8⋅2 %)	7⋅4 %	(6⋅3 %)	0⋅002	0⋅36	<0⋅001
Meat and seafood	21⋅4 %	(11⋅5 %)	20⋅7 %	(11⋅8 %)	0⋅38	0⋅67	<0⋅001
Nuts and seeds	2⋅3 %	(4⋅1 %)	2⋅5 %	(3⋅1 %)	0⋅56	0⋅58	<0⋅001
Pasta, rice and grains	1⋅6 %	(2⋅2 %)	1⋅9 %	(2⋅8 %)	0⋅17	0⋅36	<0⋅001
Salty snacks	4⋅6 %	(3⋅9 %)	6⋅3 %	(6⋅5 %)	0⋅001	0⋅39	<0⋅001
Sauces, spreads and dips	4⋅1 %	(4⋅0 %)	4⋅0 %	(3⋅4 %)	0⋅81	0⋅50	<0⋅001
Sugar-sweetened beverages	3⋅5 %	(5⋅0 %)	4⋅3 %	(5⋅5 %)	0⋅07	0⋅56	<0⋅001
Sweets	6⋅1 %	(6⋅0 %)	6⋅5 %	(5⋅8 %)	0⋅34	0⋅59	<0⋅001
Other	4⋅0 %	(5⋅0 %)	3⋅8 %	(4⋅2 %)	0⋅72	0⋅51	<0⋅001

*Note*. Definitions of food categories are provided in Supplementary Table S3. Purchases of alcohol were excluded (five items selected by three participants in the real online store) from analyses because alcohol was not available in the naturalistic online store.

## Discussion

The present study of US adults found that a naturalistic online store developed for research is highly acceptable to participants. For example, the large majority of participants in this study reported that the naturalistic store was easy to use and that they were satisfied with the number of options in the store. All participants also indicated they would be willing to participate in another study using the naturalistic online store. Moreover, participants rated the naturalistic online store similarly to the real online store, suggesting that the naturalistic store does not have major acceptability shortcomings relative to conducting studies with a real online grocer. Acceptability and feasibility measures in the present study also compared favourably with reactions to other naturalistic grocery stores used for research^(21,22)^.

Results also suggested that naturalistic store effectively mimicked a real online shopping experience. First, nearly all participants reported that the naturalistic online store felt like a real store and that their purchases in this store were similar to the groceries they usually buy. Second, participants appeared to behave in similar ways in the naturalistic online store as they do at real-world grocers. In our study, participants selected similar foods and beverages in the naturalistic online store as they did in the real online store, with correlations in spending on food categories in the moderate-to-strong range (correlations of 0⋅36–0⋅67) and few significant differences between stores. Importantly, although the present study assessed hypothetical purchases, participants’ food and beverage selections in the naturalistic store also mirrored nationally representative data on real-world grocery purchases. For example, when simulating their usual weekly shopping trip, participants in this study purchased a mean of $139 from the naturalistic online, which is within the range of average weekly spending on food-at-home among households with and without children ($106–160)^(23)^. Although differences in food categories examined between this study and national data preclude comprehensive comparisons, when these comparisons were possible, purchases in the naturalistic online store aligned well with national estimates. For example, fruit and vegetable purchases comprised 20⋅0% of total spending in the naturalistic online store, very similar to the estimated 19⋅8% of total at-home food expenditures on fruits and vegetables among households of similar size in the nationally representative Consumer Expenditure Surveys (CES)^(24)^. Likewise, spending on meat and seafood (21% in this study *v*. 21% in CES), dairy (7% *v*. 9%), eggs (2% *v*. 1%) and cereal (2% *v*. 4%) in the naturalistic store were all similar in this study compared with national estimates^(24)^. Together, these results suggest that the naturalistic online store could offer an ecologically valid setting for nutrition research.

The strengths of the present study include the use of a novel online grocery store resembling a real online grocer and the diverse sample. Limitations include that all participants had shopped for groceries online in the previous 12 months, so results may not generalise to consumers who never shop online. However, online grocery shopping has risen dramatically in recent years and is projected to comprise 20% of all grocery sales by 2024–2026^(1)^. Another limitation is that participants did not purchase their selections. This did not differ between the naturalistic and real online stores, but selections in this study could differ from real-stakes purchases. However, selections in this study aligned closely with national data on food purchases from the CES, perhaps lessening this concern. Future research will establish the feasibility of delivering groceries from naturalistic online grocery stores.

## Conclusions

Online grocery shopping is increasingly common, creating an urgent need to understand the impact of nutrition policies and interventions in online settings. Naturalistic online grocery stores offer a promising avenue for research studies seeking to evaluate how nutrition interventions affect online food purchasing behaviour.

## Supplementary material

For supplementary material accompanying this paper visit https://doi.org/10.1017/jns.2023.21. click here to view supplementary material
