# Supplementary material for: Consumer behaviour and experiences in a naturalistic online grocery store: implications for nutrition research
Source: J Nutr Sci. 2023 Mar 14;12:e36. doi: 10.1017/jns.2023.21 (PMC10052388; doi:10.1017/jns.2023.21)
Supplement: Supplementary file 1 [file S2048679023000216sup001.docx]

**Consumer Behavior and Experiences in a Naturalistic Online Grocery Store: Implications for Nutrition Research**

**Online Supplemental Materials**

**Supplemental Figure 1.** Participant flow diagram

**Supplemental Table 1.** Survey items used for eligibility screening

**Supplemental Table 2.** Survey items used to assess process evaluation and demographic variables

**Supplemental Table 3.** Food and beverage categories examined in analyses

**Supplemental Table 4.** Absolute expenditures by food category and store, *n*=144 US adults

**Supplemental Figure 1.** Participant flow diagram


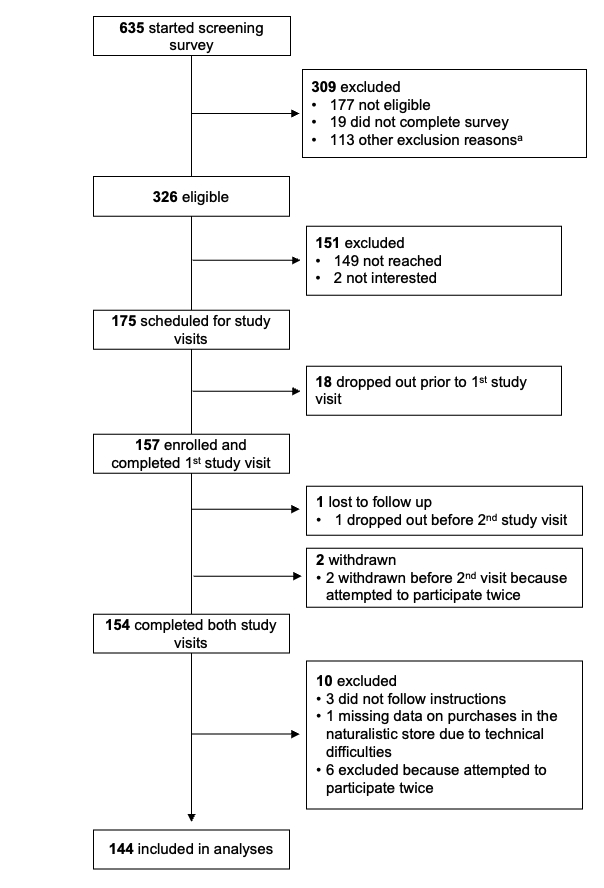


^a^Other exclusion reasons included being duplicate responses; completing the survey from an IP address outside of the US; providing contradictory identifying information; and completing the survey after it was no longer publicly posted online, suggesting the respondent may have already completed the survey at an earlier date.

**Supplemental Table 1.** Survey items used for eligibility screening

| **Variable** | **Item**  **[programming notes]** | **Response scale** |
| --- | --- | --- |
| **Consent** |  |  |
| Consent | **[Present electronic consent form]**  **[page break]** |  |
| **Demographics** |  |  |
| Prompt | **We will ask a few questions to find out whether you are eligible for our study of online grocery shopping. There are no right or wrong answers.**  **[page break]** |  |
| Age | How old are you in years? | [numeric free response, cap at 115] |
| Frequency of online grocery shopping | In the last 12 months, how often did you shop for groceries online? This includes any groceries you ordered online from grocery stores, superstores like Walmart, or online shopping websites like AmazonFresh or Instacart. These groceries could be for pickup or delivery, as long as you ordered them online. | 0=Never  1=1 time per month or less  2=2-3 times per month  3=1 time per week  4=More than 1 time per week |
| Medium used for online grocery shopping | How do you usually shop for groceries online? Select the option you use most often. | 1=Website on a computer  2=Website on a cellphone  3=Website on a tablet  4=Mobile app on a cellphone  5=Mobile app on a tablet  6=Other: [free response box] |
| Amount of household’s shopping participant does | How much of your household’s grocery shopping do you do? | 1=None  2=Less than half  3=About half  4=More than half  5=All |
| Computer access | Do you have access to a laptop or desktop computer with internet that you can use for completing a research study? | 1=Yes  0=No |
| Household size | How many people live with you in your household, including you? Only include people who live in your household at least half the time. | **[force response]**  # of people [restricted to 1-20, whole numbers] |
| Income in %s of the Federal Poverty Level | **[Ask only if household size=1]**  Which of the following categories best describes your total household income in the last 12 months? | **[force response]**  1=Less than $19,140  2=Between $19,140 and $25,519  3=Between $25,520 and $31,899  4=Between $31,900 and $38,279  5=$38,280 or more |
| Income in %s of the Federal Poverty Level | **[Ask only if household size=2]**  Which of the following categories best describes your total household income in the last 12 months? | **[force response]**  1=Less than $25,860  2=Between $25,860 and $34,479  3=Between $34,480 and $43,099  4=Between $43,100 and $51,719  5=$51,720 or more |
| Income in %s of the Federal Poverty Level | **[Ask only if household size=3]**  Which of the following categories best describes your total household income in the last 12 months? | **[force response]**  1=Less than $32,580  2=Between $32,580 and $43,439  3=Between $43,440 and $54,299  4=Between $54,300 and $65,159  5=$65,160 or more |
| Income in %s of the Federal Poverty Level | **[Ask only if household size=4]**  Which of the following categories best describes your total household income in the last 12 months? | **[force response]**  1=Less than $39,300  2=Between $39,300 and $52,399  3=Between $52,400 and $65,499  4=Between $65,500 and $78,599  5=$78,600 or more |
| Income in %s of the Federal Poverty Level | **[Ask only if household size=5]**  Which of the following categories best describes your total household income in the last 12 months? | **[force response]**  1=Less than $46,020  2=Between $46,020 and $61,359  3=Between $61,360 and $76,699  4=Between $76,700 and $92,039  5=$92,040 or more |
| Income in %s of the Federal Poverty Level | **[Ask only if household size=6]**  Which of the following categories best describes your total household income in the last 12 months? | **[force response]**  1=Less than $52,740  2=Between $52,740 and $70,319  3=Between $70,320 and $87,899  4=Between $87,900 and $105,479  5=$105,480 or more |
| Income in %s of the Federal Poverty Level | **[Ask only if household size=7]**  Which of the following categories best describes your total household income in the last 12 months? | **[force response]**  1=Less than $59,460  2=Between $59,460 and $79,279  3=Between $79,280 and $99,099  4=Between $99,100 and $118,919  5=$118,920 or more |
| Income in %s of the Federal Poverty Level | **[Ask only if household size=8]**  Which of the following categories best describes your total household income in the last 12 months? | **[force response]**  1=Less than $66,180  2=Between $66,180 and $88,239  3=Between $88,240 and $110,299  4=Between $110,300 and $132,359  5=$132,360 or more |
| Income in %s of the Federal Poverty Level | **[Ask only if household size=9]**  Which of the following categories best describes your total household income in the last 12 months? | **[force response]**  1=Less than $72,900  2=Between $72,900 and $97,199  3=Between $97,200 and $121,499  4=Between $121,500 and $145,799  5=$145,800 or more |
| Income in %s of the Federal Poverty Level | **[Ask only if household size=10]**  Which of the following categories best describes your total household income in the last 12 months? | **[force response]**  1=Less than $79,620  2=Between $79,620 and $106,159  3=Between $106,160 and $132,699  4=Between $132,700 and $159,239  5=$159,240 or more |
| Income in %s of the Federal Poverty Level | **[Ask only if household size=11]**  Which of the following categories best describes your total household income in the last 12 months? | **[force response]**  1=Less than $86,340  2=Between $86,340 and $115,119  3=Between $115,120 and $143,899  4=Between $143,900 and $172,679  5=$172,680 or more |
| Income in %s of the Federal Poverty Level | **[Ask only if household size=12]**  Which of the following categories best describes your total household income in the last 12 months? | **[force response]**  1=Less than $93,060  2=Between $93,060 and $124,079  3=Between $124,080 and $155,099  4=Between $155,100 and $186,119  5=$186,120 or more |
| Income in %s of the Federal Poverty Level | **[Ask only if household size=13]**  Which of the following categories best describes your total household income in the last 12 months? | **[force response]**  1=Less than $99,780  2=Between $99,780 and $133,039  3=Between $133,040 and $166,299  4=Between $166,300 and $199,559  5=$199,560 or more |
| Income in %s of the Federal Poverty Level | **[Ask only if household size=14]**  Which of the following categories best describes your total household income in the last 12 months? | **[force response]**  1=Less than $106,500  2=Between $106,500 and $141,999  3=Between $142,000 and $177,499  4=Between $177,500 and $212,999  5=$213,000 or more |
| Income in %s of the Federal Poverty Level | **[Ask only if household size=15]**  Which of the following categories best describes your total household income in the last 12 months? | **[force response]**  1=Less than $113,220  2=Between $113,220 and $150,959  3=Between $150,960 and $188,699  4=Between $188,700 and $226,439  5=$226,440 or more |
| Income in %s of the Federal Poverty Level | **[Ask only if household size=16]**  Which of the following categories best describes your total household income in the last 12 months? | **[force response]**  1=Less than $119,940  2=Between $119,940 and $159,919  3=Between $159,920 and $199,899  4=Between $199,900 and $239,879  5=$239,880 or more |
| Income in %s of the Federal Poverty Level | **[Ask only if household size=17]**  Which of the following categories best describes your total household income in the last 12 months? | **[force response]**  1=Less than $126,660  2=Between $126,660 and $168,879  3=Between $168,880 and $211,099  4=Between $211,100 and $253,319  5=$253,320 or more |
| Income in %s of the Federal Poverty Level | **[Ask only if household size=18]**  Which of the following categories best describes your total household income in the last 12 months? | **[force response]**  1=Less than $133,380  2=Between $133,380 and $177,839  3=Between $177,840 and $222,299  4=Between $222,300 and $266,759  5=$266,760 or more |
| Income in %s of the Federal Poverty Level | **[Ask only if household size=19]**  Which of the following categories best describes your total household income in the last 12 months? | **[force response]**  1=Less than $140,100  2=Between $140,100 and $186,799  3=Between $186,800 and $233,499  4=Between $233,500 and $280,199  5=$280,200 or more |
| Income in %s of the Federal Poverty Level | **[Ask only if household size=20]**  Which of the following categories best describes your total household income in the last 12 months? | **[force response]**  1=Less than $146,820  2=Between $146,820 and $195,759  3=Between $195,760 and $244,699  4=Between $244,700 and $293,639  5=$293,640 or more |

**Supplemental Table 2.** Survey items used to assess process evaluation and demographic variables

| **Variable**  **(Source)** | **Item**  **[programming notes]** | **Response scale** | **Asked at study visit 1?** | **Asked at study visit 2?** |
| --- | --- | --- | --- | --- |
| **Process evaluation** |  |  |  |  |
| Prompt | The next questions are about the online grocery store. |  | X | X |
| Overall ease of using this store | Overall, how difficult or easy was it to use this online grocery store? | 1=Very difficult  2=Difficult  3=Neither difficult nor easy  4=Easy  5=Very easy | X | X |
| Ability to find items on list | How many items on your list were you able to find? | 1=None  2=Some things  3=Most things  4=Almost everything  5=Everything | X | X |
| Most difficult aspects of shopping in this store | Which of the following, if any, made it difficult to shop in this online grocery store? | 1=Nothing about using this store was difficult [make answer exclusive]  2=Finding the types of foods and beverages I wanted  3=Finding the specific brands I wanted  4=Staying within my usual budget  5=Using the search bar  6=Getting the pages to load  7=Adding items to my cart  8=Removing items from my cart  9=Finding product information (e.g., nutrition facts, ingredients)  10=Selecting the quantity of items I wanted  11=Navigating on the website  12=Checking out  13=Other: ____ | X | X |
| Willingness to participate in another research study using this store | Imagine you have been invited to participate in another research study in which you selected foods and beverages in this online grocery store. You would get up to $40 for participating. How willing would you be to participate in the study?  **[page break]** | 1=Definitely not willing  2=Probably not willing  3=Probably willing  4=Definitely willing | X | X |
| Prompt | Say whether you agree or disagree with the following statements. |  | X | X |
| Ease of finding everything was looking for    ^18^ | It was easy to find everything I was looking for in this online grocery store. | 1=Strongly disagree  2=Somewhat disagree  3=Neither agree nor disagree  4=Somewhat agree  5=Strongly agree | X | X |
| Satisfaction with number of options available  ^18^ | I was satisfied with the number of options in this online grocery store. | 1=Strongly disagree  2=Somewhat disagree  3=Neither agree nor disagree  4=Somewhat agree  5=Strongly agree | X | X |
| Would choose similar products in the real-world | If I completed this shopping task in the real world, I would pick products similar to the products I chose in this study. | 1=Strongly disagree  2=Somewhat disagree  3=Neither agree nor disagree  4=Somewhat agree  5=Strongly agree | X | X |
| Choices were similar to usual grocery purchases | The foods I selected in this online grocery store were similar to my usual grocery purchases. | 1=Strongly disagree  2=Somewhat disagree  3=Neither agree nor disagree  4=Somewhat agree  5=Strongly agree | X | X |
| Store felt like a real store  ^18^ | This online grocery store felt like a real online grocery store.      **[page break]** | 1=Strongly disagree  2=Somewhat disagree  3=Neither agree nor disagree  4=Somewhat agree  5=Strongly agree | X | X |
| Why were selections different than usual purchases? | [only display if participant indicated they disagreed that their choices were similar to usual purchases]  You indicated that you disagreed that the foods you selected in this store were similar to your usual grocery purchases. Why were your selections in this store different from your usual purchases? | [free response text] | X | X |
| What would make this store feel more like a real store? | [only display if participant indicated they disagreed that the store felt like a real store]  You indicated that you disagreed that this store felt like a real online grocery store. What would make this store feel more like a real online grocery store?  **[page break]** | [free response text] | X | X |
| Demographics |  |  |  |  |
| Introduction to demographics | We are asking the questions in the next section to better understand who completed this survey. |  | X | X |
| Gender | Which gender do you most identify with? | 1=Woman  2=Man  3=Non-binary  4=Prefer to self-describe:_____ | X |  |
| Educational attainment | What is the highest degree or level of school you have completed? | 1=Less than high school  2=High school graduate (or GED)  3=Some college or technical school  4=Associate’s degree  5=Bachelor’s degree  6=Graduate or professional degree | X |  |
| Latino(a) or Hispanic ethnicity | Are you of Hispanic, Latino, or Spanish origin? | 1=Yes  0=No | X |  |
| Race | What is your race? (Check all that apply).  **[page break]** | [Check all that apply]  1=White  2=Black or African American  3=American Indian or Alaska Native  4=Asian  5=Native Hawaiian or Other Pacific Islander  6=Some other race: | X |  |
| Age | How old are you in years? | [numeric free response, cap at 115] |  | X |
| Usual weekly grocery budget | About how much does your household spend on groceries in a typical week?  Include any foods you or other members of your household buy from stores in a usual week (i.e., without a holiday or special occasion). Do NOT include foods you buy from restaurants. | 1=$50 or less  2=$51-$75  3=$76-$100  4=$101-$125  5=$126-$150  6=$151-$175  7=$176-$200  8=$201-$250  9=$251-$300  10=$301-$400  11=More than $400 (enter approximate amount) |  | X |
| Use of online grocery store features | Which of the following features, if any, do you use when shopping for groceries online? (Check all apply) | [check all that apply, randomize responses]  1=Search bar  2=Sort function  3=Product ratings  4=Best sellers  5=Nutrition information  6=Shopping lists  7=Recipes  8=Past purchases list/Purchase history  9=Filtering  10=Health ratings like Guiding Stars  11=Pages for specific diets (e.g., keto, plant-based, gluten-free)  12=None of these [make exclusive, always last] |  | X |
| Perceived dietary quality  ^26^ | In general, how healthy is your overall diet? Would you say… | 1=Poor  2=Fair  3=Good  4=Very good  5=Excellent |  | X |
| Prompt | The next questions are about the Nutrition Facts label.  **[Insert image of Nutrition Facts label].** |  |  | X |
| Use of Nutrition Facts Label online  ^27^ | When you are shopping for groceries online, how often do you use the Nutrition Facts label when deciding to buy a food product? | 1=Never  2=Rarely  3=Sometimes  4=Most of the time  5=Always |  | X |
| Use of Nutrition Facts Label in store  ^27^ | When you are shopping for groceries at a store, how often do you use the Nutrition Facts label when deciding to buy a food product?  **[page break]** | 1=Never  2=Rarely  3=Sometimes  4=Most of the time  5=Always |  | X |
| Household size | How many people live with you in your household, including you? Only include people who live in your household at least half the time. | [free response, 1-20] |  | X |
| Number of children | How many children (ages 0-18) currently live in your household? | [#, restricted to 0-15, whole numbers] |  | X |
| Household income (10-level) | Which of the following categories best describes your total household income in the last 12 months?  **[page break]** | 1=Less than $10,000  2=$10,000 to $14,999  3=$15,000 to $24,999  4=$25,000 to $34,999  5=$35,000 to $49,999  6=$50,000 to $74,999  7=$75,000 to $99,999  8=$100,000 to $149,999  9=$150,000 to $199,999  10=$200,000 or more |  | X |
| **Closure** |  |  |  |  |
| Most difficult part of the study | In this study, you completed two online study visits with a member of the study team. At each visit, you shopped for groceries in an online store and answered questions in an online survey. Thinking about the study as a whole, what was the most difficult part of the study?  **[page break]** | **[check all that apply]**  1=Nothing about this study was difficult [make exclusive]  2=Finding appointment times that worked for my schedule  3=Remembering to go to my study appointments  4=Having to go to 2 appointments  5=Internet or computer issues  6=Shopping in Walmart  7=Shopping in Lola’s  8=Answering the survey questions  9=Other:______ |  | X |
| Free response comments | Is there anything else you want to tell us?  **[page break]** | [Open ended] | X | X |
| Closure | You have now finished this survey. Thank you for your participation! |  | X | X |

**Supplemental Table 3.** Food and beverage categories examined in analyses

| **Category name** | **Description** |
| --- | --- |
| Bread | Breads, bagels, buns, rolls, flatbreads, tortillas |
| Cereal | Cold cereals, granola, oatmeal |
| Dairy | Milk, cream, half & half, cream, coffee creamers, yogurt, cheese, sour cream, sweetened milk, and non-dairy (i.e., vegan) alternatives for these products; excludes butter and ice cream |
| Eggs | Eggs and egg substitutes; excludes egg-based entrees |
| Entrees | Frozen or fresh meals or meal kits (including breakfast and dinner kits as well as waffles and pancake mixes), soups, pizzas, salad kits, sandwiches & wraps, Lunchables and snack packs, sushi, party platters, pasta dishes, frozen appetizers/sides; excludes raw meat (e.g., ground beef) |
| Fruits, vegetables, and legumes | Fresh, canned, frozen, or dried fruits, vegetables (including potatoes, tomato paste, and tomato sauce), and beans/legumes |
| Low-calorie drinks and 100% juice | Sodas, fruit drinks, sports drinks, flavored waters, and coffee/tea that are unsweetened, sweetened with non-caloric sweeteners, or sweetened with caloric sweeteners but contain <2 calories per fluid ounce; 100% juice without added sweeteners (including lemon and lime juice) |
| Meat and seafood | Beef, pork, lamb, poultry, fish, shellfish and lobster, and meat alternatives, including canned meat and seafoods and processed meats (e.g., pepperoni, salami); excludes jerky and rinds |
| Nuts and seeds | Peanuts, almonds, pistachios, etc. (including roasted or salted nuts without any other ingredients); nut butters (including sweetened nut butters) |
| Pasta, rice, and grains | Dry pasta, rice, quinoa, couscous, grits; excludes canned pasta entrees (e.g., canned ravioli) |
| Salty snacks | Chips, pretzels, crackers, popcorn, trail mix, jerky and pork rinds |
| Sauces, spreads, dips | Chip dip, salsa, guacamole, hummus, butter, margarine, cream cheese, jelly, jam, syrup, honey, ketchup, mustard, marinades, sauces, mayo, salad dressings |
| Sugar-sweetened beverages | Calorically sweetened soda, fruit drinks, sports drinks, flavored waters, and coffee/tea containing >2 calories per fluid ounce |
| Sweets | Muffins, croissants, brownies, cakes, cupcakes, cookies, pastries, pies, or tarts, chocolate and other candy, ice cream, Jell-o, pudding, frosting, snack bars (granola bars, energy bars, etc.), toaster pastries, and whipped topping; includes mixes to make these products (e.g., boxed brownie mix) |
| Other | All other products, including baby foods, gum, herbs, spices, baking supplies (vanilla, evaporated or sweetened condensed milk, yeast, etc.), breadcrumbs, cooking staples (oils, flour, sugar/sugar substitutes, etc.), yogurt starters, and any other product not otherwise categorized |

*Note*. Alcohol was excluded from analyses because no alcohol was available in the naturalistic online store.

**Supplemental Table 4.** Absolute expenditures by food category and store, *n*=144 US adults

|  | **Absolute expenditures ($)** | | | |
| --- | --- | --- | --- | --- |
|  | **Naturalistic Online Store**  **Mean (SD)** | | **Real Online Store**  **Mean (SD)** | |
| Bread | 4.82 | (4.03) | 5.65 | (6.03) |
| Cereal | 3.19 | (4.19) | 3.83 | (4.62) |
| Dairy | 8.78 | (6.87) | 9.37 | (7.96) |
| Eggs | 1.94 | (1.81) | 2.63 | (2.06) |
| Frozen and packaged entrees | 12.39 | (18.30) | 15.11 | (22.46) |
| Fruits, vegetables, and legumes | 26.57 | (20.93) | 27.42 | (17.92) |
| Low-calorie drinks and 100% juice | 13.65 | (14.83) | 11.36 | (10.28) |
| Meat and seafood | 30.24 | (24.49) | 31.71 | (23.89) |
| Nuts and seeds | 3.19 | (4.96) | 3.77 | (4.91) |
| Pasta, rice, and grains | 2.36 | (4.02) | 3.34 | (5.87) |
| Salty snacks | 6.60 | (7.12) | 10.21 | (11.08) |
| Sauces, spreads, and dips | 5.99 | (6.56) | 6.69 | (7.27) |
| Sugar-sweetened beverages | 4.44 | (6.02) | 6.76 | (9.23) |
| Sweets | 8.37 | (8.49) | 11.18 | (12.66) |
| Other | 6.51 | (9.57) | 7.01 | (9.89) |
| Total | 139.02 | (83.96) | 156.04 | (85.75) |

*Note*. Definitions of food categories are provided in Supplemental Table 3. Purchases of alcohol were excluded (5 items selected by 3 participants in the real online store) from analyses because alcohol was not available in the naturalistic online store.
